# Fabrication of Novel Agrowaste (Banana and Potato Peels)-Based Biochar/TiO_2_ Nanocomposite for Adsorption of Cr(VI), Statistical Optimization via RSM Approach

**DOI:** 10.3390/polym14132644

**Published:** 2022-06-29

**Authors:** Aamna Ashfaq, Raziya Nadeem, Hongyu Gong, Umer Rashid, Saima Noreen, Shafique ur Rehman, Zubair Ahmed, Muhammad Adil, Nayab Akhtar, Muhammad Zeeshan Ashfaq, Fahad A. Alharthi, Elham Ahmed Kazerooni

**Affiliations:** 1 Key Laboratory for Liquid-Solid Structural Evolution & Processing of Materials, Ministry of Education, School of Materials Science and Engineering, Shandong University, Jinan 250061, China; amnaashfaq9@yahoo.com (A.A.); hygong@email.sdu.edu.cn (H.G.); zee4194202@gmail.com (M.Z.A.); 2 Department of Chemistry, University of Agriculture, Faisalabad 38040, Pakistan; saima_bashir03@yahoo.com (S.N.); shafiq.urrehman@uaf.edu.pk (S.u.R.); 2015ag847@uaf.edu.pk (Z.A.); muhammadadil507@gmail.com (M.A.); 3 Institute of Nanoscience and Nanotechnology (ION2), Universiti Putra Malaysia, Serdang 43400, Selangor, Malaysia; 4 Department of Zoology, Lahore College for Women University, Lahore 54000, Pakistan; nayabzeeshan7863@gmail.com; 5 Chemistry Department, College of Science, King Saud University, Riyadh 1145, Saudi Arabia; fharthi@ksu.edu.sa; 6 Department of Applied Biosciences, Kyungpook National University, Daegu 41566, Korea; elham.ghasemi.k@gmail.com

**Keywords:** TiO_2_ nanocomposite (TiO_2_ NC), biomass, biochar, green synthesis, kinetic model, equilibrium model

## Abstract

In this research work, a simple, efficient, and eco-friendly procedure for the biosorption of Cr(VI) ions was studied. A detailed comparative study was performed to check the adsorption efficiency of agrowaste (banana and potato peels)-based adsorbents. Firstly, mixed biosorbent was washed, dried and ground into powder, secondly, biosorbent was pyrolyzed into biochar and thirdly TiO_2_ nanocomposite (TiO_2_ NC) biosorbent was made by sonicating using prepared biochar and TiO_2_ NPs. Titanium dioxide nanoparticles (TiO_2_ NPs) were synthesized by a green method using *Psidium guajava* leaf extract. The synthesized adsorbents were characterized by SEM, EDX FT-IR, XRD and UV-visible analysis. The effect of four different factors, i.e., pH of the synthetic metallic solution, time, concentration and adsorbent dosage was studied. The optimum conditions were time (120 min), pH (3), concentration (10 ppm) and adsorbent dosage (1.0 g). The kinetic modeling showed that the adsorption of Cr(VI) ion follows a pseudo second-order mechanism and the Langmuir isotherm model was found to fit better for this study. Response surface methodology (RSM)-based optimized parameters provided optimal parameter sets that better represent the adsorption rate models. The uptake capacity of Cr(VI) from aqueous solution was found to be biomass (76.49 mg/L) ˂ biochar (86.51 mg/L) ˂ TiO_2_ NC (92.89 mg/L). It can be suggested that the produced TiO_2_ NC could possibly be an efficient biosorbent for the removal of Cr(IV).

## 1. Introduction

The prime component that is responsible for life on earth is water. It should be pollutant-free for drinking and other domestic and industrial activities. Natural resources are being contaminated with the increase in population and industrialization. A large amount of water from industries is released which leads to water pollution causing dangerous effects on plants, human and marine life. Effluents discharged from the industries include heavy metals, pharmaceutical drugs, dyes and other effluents which affect the water quality [1]. Heavy metals pollution in the aquatic environment is a big problem due to its presence, contamination in the food chain and adverse effects on ecological and human health [2]. Many physical and chemical methods are used to remove heavy metals from wastewater [3]. Ultrafiltration membrane, coagulation, reverse osmosis, ozonation and photocatalytic degradation are methods to treat wastewater [2,3]. However, they have some limitations, e.g., being costly, time-consuming and producing secondary by-products. Adsorption is an appropriate method which is an effective, environment-friendly and economic method for eliminating toxic heavy metal ions from wastewater effluents [4]. This method is easy to plan and apply and permits us to generate extremely good-quality treated waste material. The adsorbents may be activated carbons, fly ash, clay minerals, zeolites, industrial waste, agricultural waste, biomass and polymeric materials, etc. [5]. Chromium is a model pollutant in this study, it badly affects the aquatic system. The permissible limit for chromium recommended by the environmental protection agency is 50 µg/L [6]. Chromium affects the body organs, when it enters through the lungs it causes chronic bronchitis, irritation, rhinitis, pharyngitis, hyperemia and mucous nasal membrane ulcers. Skin exposure causes irritation or dermatitis due to allergy. Chromium also causes cancer in the respiratory tract, mainly lung, sinus and nasal cancer. Other mild effects include weakness, eye irritation, dizziness, erosion or discoloration of teeth [6].

Green synthesis of nanomaterials requires no need for high pressure and temperature, using nontoxic materials and low cost leads to environmentally friendly products. Plant extracts have been used as stabilizers, capping agents and reducing agents [5]. Nanoparticles (NPs) have appeared as an excellent choice for dealing with the global issue of water pollution. Recently, the synthesis of nanoparticles with precise morphologies and properties has gained a lot of interest. The large surface area and unique physical, chemical and optical properties make them good adsorbents for a wide range of pollutants [7]. TiO_2_ NPs have high redox potential, low toxicity and adjustable optoelectronic properties. They have several applications such as antifogging mirrors, solar cells and self-cleaning antibacterial coatings [8,9]. TiO_2_ NPs are one of the most important photocatalytic materials because they are a wide-bandgap semiconductor material (~3.2 eV) suitable only to absorb UV light and, hence, photogenerate charge carriers, electrons in the conduction band and holes in the valence band [10,11,12]. They are novel and active photocatalysts for wastewater treatment due to their high stability, low cost and high efficiency. Heterogeneous photocatalysis using TiO_2_ NPs has been an interesting method in wastewater treatment due to its ability to degrade organic contaminants [13]. 

Potato (*Solanum tuberosum*) peels contain vitamins, starch, non-starch polysaccharide, protein, acid-soluble/insoluble lignin, lipids and ash. They are reported as adsorbents for the removal of several heavy metals in literature [14]. Banana (*Musa acuminate*) peels have an affinity for heavy metal adsorption because of various potential groups such as amino, alkoxy hydroxyl and carbonyl groups [15]. Peels of bananas were reported to be used as adsorbents for several heavy metals [16]. Dried and ground biomass is used in the present study, 1:1 of both banana peels and potato peels is used to prepare mixed biomass (MBM) [17].

Biochar is a material that contains an abundant amount of carbon, and it is gained from biomass, it is preferable because it is easily available, has a wide surface area, a large amount of functional groups present on the surface area, a possible conversion process and eco-friendly behavior [18]. Synthesis of functional biochar of enhanced surface characteristics has great attention and is also a great challenge for the development of the adsorption process. Two main factors that greatly affect the surface property of biochar are biomass type and its carbonization [19]. Nanocomposite is a matrix in which nanoparticles are loaded due to which surface area and stability is increased along with the properties of nanomaterials such as thermal conductivity, strength, surface area, etc. [20]. 

In the present research work, TiO_2_ NPs were synthesized using *Psidium guava* leaf extract and TTIP, then these TiO_2_ NPs were loaded on a matrix to prepare the nanocomposite. This work aims to compare adsorption capacity mg/g of mixed biomass (MBM) (grounded banana and potato peels), biochar (BC) of prepared biomass and TiO_2_/agrowaste-based biochar nanocomposite (TiO_2_ NC), which were made by using *Psidium guajava* leaf extract for the synthesis of nanoparticles and biochar of MBM as a matrix. Different parameters such as contact time, pH, initial concentration and adsorbent dosage were studied. Furthermore, the kinetic data and equilibrium of biosorption were tested using isotherm models (Langmuir and Freundlich) and two kinetic models (pseudo first-order and pseudo second-order).

## 2. Materials and Methods

### 2.1. Materials and Chemicals

Titanium tetra hydroxide (TTIP), sodium hydroxide (NaOH), hydrochloric acid (HCl), potassium dichromate (K_2_Cr_2_O_7_), acetone and ethanol were purchased from Merck, Chemical Company, Hamburg, Germany. All chemicals involved in this study are analytical reagent grade, and solutions were all prepared with deionized water. All dilutions have been made in double-distilled water. Glassware was cleaned and rinsed with double-distilled water. They were dried in an electric oven at 50–55 °C.

### 2.2. Preparation of Adsorbate Solution

A stock solution of hexavalent chromium Cr(VI) was prepared by dissolving 5.65 g of potassium dichromate (K_2_Cr_2_O_7_) in 1000 mL of distilled water. From the 1000 mg/L stock solution, 10 mL was diluted to 100 mL which gives a 100 mg/L (100 ppm) working solution. Experimental solutions of desired concentration were then obtained by successive dilutions. The initial pH measurements were performed by pH instrument and were calibrated using the buffer solutions of pH values. The pH of the synthetic contaminated water was modified by the addition of dilute NaOH and HCl solutions.

### 2.3. Synthesis of Biosorbents

In this work, three biosorbents were made for comparative study. Firstly, mixed biomass (MBM) was prepared by collecting peels of banana (*Musa acuminata*) and potato (*Solanum tuberosome*) from local market and household waste. They were washed properly and rinsed with deionized water to remove all dust particles and then dried in sunlight for a week. The dried peels of agrowaste (banana and potato peels) were ground into a fine powder with a pestle and mortar then it was passed through the sieve to get a particle size of ~0.5 mm. Then they were stored in hermetic plastic bags for further use [21]. Secondly, the MBM was pyrolyzed at 550 °C for 2 h at a heating rate of 2 °C min^−1^. The resulting biosorbent is biochar (BC) [22]. Lastly, to prepare TiO_2_ NPs had been prepared by green synthesis method using Titanium Tetraisopropoxide (TTIP) and extract of guava leaves [23]. The plausible mechanism for synthesis of TiO_2_ NPs could be following:

Hydrolysis:

Ti(OCH(CH_3_)_2_)_4_ + 4H_2_O → Ti(OH)_4_ + 4(CH_2_)_2_CHOH (1)

Condensation:

Ti(OH)_4_ → TiO_2_·*x*H_2_O + (2 − *x*)H_2_O (2)

Crystallisation via calcination:

TiO_2_·*x*H_2_O → TiO_2_ (3)

where *x* = number of water molecules.

TiO_2_ nanoparticles were formed when TTIP undergo hydrolysis and condensation. Initially, When TTIP undergoes hydrolysis, (Ti(OH)_4_) was formed as an intermediate as shown in Equation (1). Ti(OH)_4_ undergoes condensation as it is usually not stable and amorphous hydrous oxide precipitates (TiO_2_·*x*H_2_O) were formed as stated in Equation (2). The formed TiO_2_·*x*H_2_O precipitates were then subjected to the calcination process at 500 °C to remove the water molecules to form the crystalline TiO_2_ NPs as in Equation (3) [24].

### 2.4. Characterization of Biosorbents

The prepared adsorbents were characterized by different analytical techniques, the elemental composition was studied through EDX. The prepared TiO_2_ NPs were confirmed through the UV-visible spectrophotometer (UV-2250, Shimadzu, Tokyo, Japan). Then, 50 mg of BC was sonicated with ethanol and then TiO_2_ NPs were added to dispersed BC. The developed dispersed mixture was ultrasonicated for 3 h followed by drying at moderate conditions to remove the solvent. After drying, the prepared nanocomposite was deposited on a petri dish and subjected to microwave irradiation for 2 min to give better exfoliation [25]. XRD technique (BTX III Benchtop XRD Analyzer, Olympus Corporation, Tokyo, Japan) was used to find if adsorbents had crystalline structure. Particle size was calculated through Scherrer’s formula, which is given as:

(4) $$D=K\frac{\lambda }{\beta \cos\theta }$$

where, *K* is a constant, *λ* is the wavelength of radiation and *β* is the full width half maximum.

Functional groups were determined through FT-IR (Perkin Elmer FT-IR Spectrophotometer, Waltham, MA, USA). The surface morphology of biosorbents before and after the experiment was studied by SEM (Carl Zeiss AG-SUPRA 35 VP SEM, Oberkochen, Germany).

### 2.5. Optimization of Process Parameters Methodology

A batch adsorption experiment was conducted to optimize the parameters. The removal efficiency of Cr(IV) was examined by different parameters. All glassware and apparatus were washed with nitric acid and then with distilled water for removing impurities and dust particles. In this way, all types of Cr (VI) contamination were removed from the apparatus, and it was ready for further use under experimental conditions. For investigating of adsorption behavior of Cr(VI), pH values were optimized (1, 3, 5, 7, 10), as was the metal initial concentration range (10, 20, 40, 60, 80, 100 ppm). Contact time was also optimized at (30, 60, 120, 180, and 240 min). For maintaining the pH of the solution, 1M sodium hydroxide (NaOH) and hydrochloric acid (HCl) was used. After all these steps, experimental flasks were removed from the orbital shaker. The filter paper was used to filter the suspensions of nanocomposite and biomass and primary tests were performed to find out the equilibrium time. The concentration of residual Cr(VI) in samples was determined by atomic absorbance spectrophotometer. The amount of Cr(IV) removal was calculated using the following formula:

(5) $$q=\frac{\left(Ci-Ce\right)v}{Ci}x\;$$

where *C_i_* and *C_e_* are the Cr (IV) concentrations in mg/L initially and at a given time, *v* is the volume of solution (L) and *m* is the mass of the adsorbent (g).

The potential rate-controlling step and kinetic mechanism of Cr(VI) biosorption were evaluated by adsorption kinetic. The equation of the pseudo 1st-order regression model for adsorption is stated as follows:

(6) $$\log\left(qe\;\right)–\;\left(k\;1\frac{1}{0.2303}\right)t$$

where, *qe* = amount of Cr(VI), and *q* (mg/g) = amount of Cr(VI) that is adsorbed on the adsorbents at equilibrium state, t is the time to achieve the equilibrium, the unit used for these amounts is mg/g, *k*1 (min^−1^) represents the rate constant for the pseudo 1st-order reaction equation. Data of adsorption kinetics was again studied by pseudo 2nd-order kinetic model which presumes that sorption rate depends on adsorption capacity. The square of unoccupied sites is proportional to the sorption rate. It is shown as

(7) $$\frac{t}{q}=\frac{1}{k2qe2}+\frac{t}{qe}$$

where, *k*2 = equilibrium rate constant (g/mg/min). The slopes and intercept of plots *t*/*q* versus *t* were used to calculate the pseudo 2nd order rate constant *k*2 and *qe*.

Equilibrium modeling was used to define the relationship between adsorbent and adsorbate molecules in an aqueous solution. The Langmuir isotherm equation is written as

(8) $$\frac{Ci}{qe}=\frac{1}{qmax}+\frac{1}{b\;qmax}Ce$$

where, *Ce* = equilibrium concentration, *qe* = equilibrium adsorption capacity, *b* = slope and 1/*qmax* = Intercept obtained from regression equation. The linear equation of Langmuir isotherm is written as

(9) $$\frac{Ce}{qe}=\frac{1}{Xm}\mathrm{KL}\;+\frac{Ce}{Xm}$$

where, *Ce* = equilibrium concentration, *qe* = amount of lead adsorbed at equilibrium, *Xm* and KL are Langmuir constants obtained by using the linearized Langmuir equation and Langmuir parameters.

The Freundlich isotherm equation is generally written as

(10) $$qe=K\;Ce\frac{1}{n}$$

The linear equation for the Freundlich isotherm is

(11) $$Log\;qe=\log Kf+\left(\frac{1}{n}\right)\log Ce$$

where, *qe* (mg/g) = quantity of Cr (VI) adsorbed by specified amount of adsorbent at equilibrium, *Ce* (mg/L) = equilibrium concentration, *Kf* = intercept constant obtained from regression equation, and 1/*n* = slope constant obtained from regression equation [26].

The interaction and optimization of three important independent variables, i.e., pH, initial concentration of Cr(VI) and adsorbent dosage were investigated by using response surface methodology. The Box–Behnken design was applied to predict and model the complex relations between the 3 parameters (pH, concentration and adsorbent dosage). The polynomial model was calculated through Design Expert 8.0 software and the formula could be described by Equation (12):

(12) $$R\left(\%\right)=a0+\overset{n}{\underset{i=1}{\sum }}aiXi+\overset{n}{\underset{i=1}{\sum }}aiiX2\;ii+\varepsilon$$

where *a* was the coefficient; *X* was the independent variable, and ε was the random error [27].

## 3. Results and Discussion

### 3.1. Characterization of Adsorbent

#### 3.1.1. UV Analysis of TiO_2_ NPs

**Figure 1:**
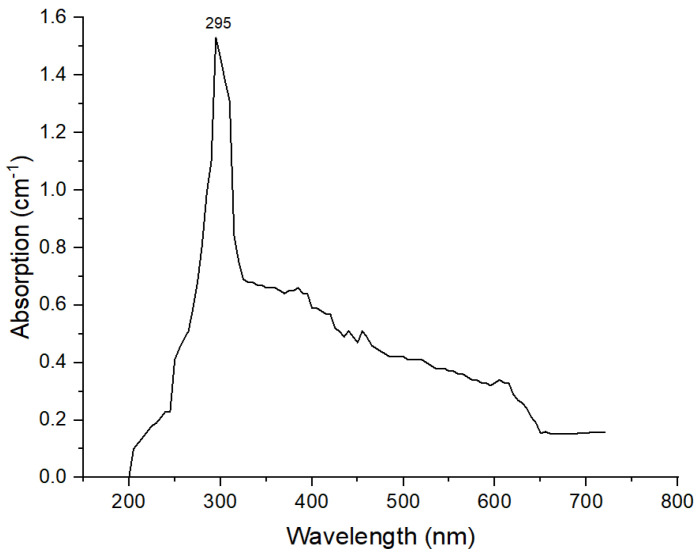
UV spectra of TiO_2_ NPs.

Synthesis was confirmed by a UV/visible spectroscopy peak of maximum absorption observed between 290 and 310 nm. These results confirmed the synthesis of TiO_2_ NPs. A maximum absorption peak was observed at 295 nm, the UV spectrum is shown in Figure 1. The results agreed with previously reported literature, where the peak was observed at 310 nm [28,29].

#### 3.1.2. Scanning Electron Microscopy (SEM) and EDX Analysis

**Figure 2:**
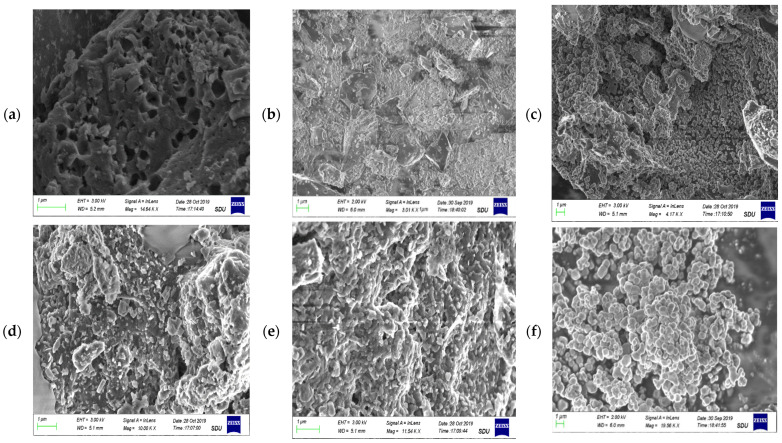
SEM micrograph of (**a**) biomass; (**b**) biochar; (**c**) TiO_2_ NC; (**d**) lead-loaded biomass; (**e**) lead-loaded biochar; and (**f**) lead-loaded TiO_2_ NC.

The morphology of prepared adsorbents is described in Figure 2. SEM micrographs of the MBM, BC and TiO_2_ NC are shown in Figure 2a–c which shows the bulky and smooth morphology of the bare biosorbents. MBM has hollow spaces, while biochar and TiO_2_ NC have agglomerated structures, Figure 2d–f shows Cr(VI) loaded biosorbents. A clear difference can be seen between the surface morphology of Cr(VI) loaded and unloaded biosorbents at the same magnification.

**Figure 3:**
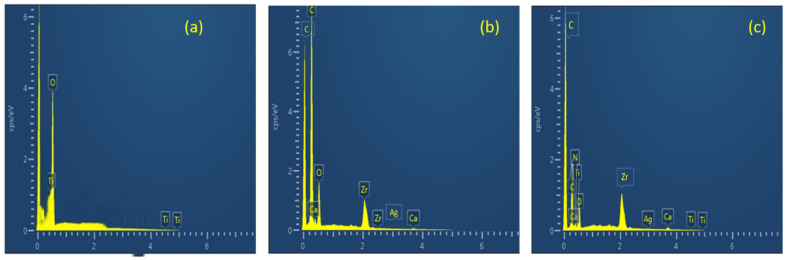
EDX spectra of (**a**) TiO_2_ NP; (**b**) biomass; and (**c**) TiO_2_ NC.

The elemental composition of prepared biosorbents was analyzed by EDX analysis. The higher the number of a particular element, the higher its presence in that point or area of interest. The EDX spectrum of TiO_2_ NP is shown in Figure 3a. It is indicating strong Ti peaks at 0.7, 4.6 and 4.9 kev (1.2 cps/ev). The peak for oxygen also appeared at 0.5 kev (4.1 cps/ev). Figure 3b represents the elemental composition of MBM. Zr, Ca and O can be seen from the spectra. Figure 3c shows the spectra of TiO_2_ NC. Table 1 shows the weight (%) and atomic (%) of the constituent elements of TiO_2_ NP, MBM and TiO_2_ NC.

#### 3.1.3. FT-IR Spectroscopy

**Figure 4:**
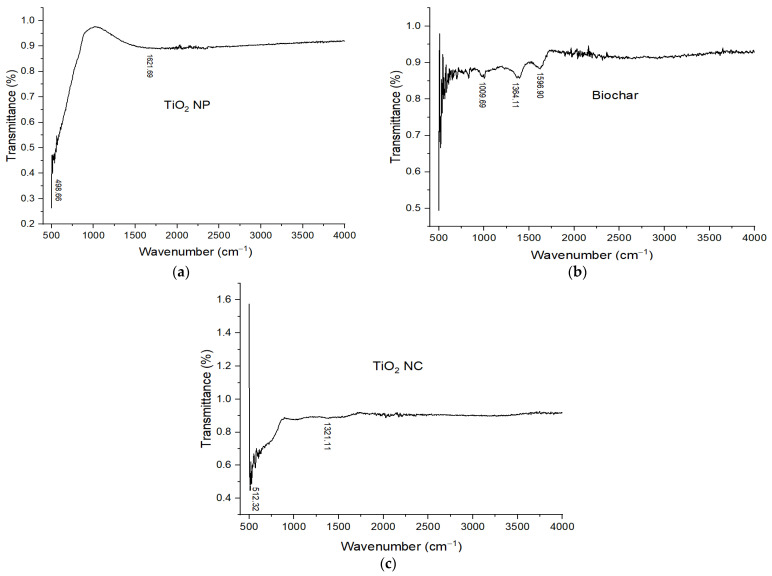
FT-IR spectra of (**a**) TiO_2_ NP; (**b**) biochar; and (**c**) TiO_2_ nanocomposite.

FT-IR is a nondestructive analytical technique used for identifying the chemical bonds in a matter with the interaction of infrared (IR) radiation. When the sample is exposed to IR light, some light is reflected but a large portion of IR light is absorbed (which causes the molecules to vibrate) by the sample. A molecular fingerprint of the sample is created by the transmitted light and the resulting spectrum represents absorption or transmission vs wavenumber. Every sample has a different composition and unique arrangement of atoms. FT-IR is helpful for the qualitative and quantitative analysis of organic and inorganic samples [30]. The molecular fingerprint of materials was examined through Fourier transform infrared spectroscopy, allowing close examination of a sample’s chemical composition. FT-IR spectra of TiO_2_ NP are shown in Figure 4a. From the spectra, no peaks are observed in the functional group because there is no functional group present in the prepared sample. The peaks are present at 498 cm^−1^ to 550 cm^−1^ these peaks are of O–Ti–O and Ti–O bending vibrations. These peaks are present in the fingerprint region of the spectra. Broad peaks are present at 1621.69 cm^−1^, these peaks show the Ti–OH stretching vibration.

The FT-IR spectrum of BC is shown in Figure 4b. The absorption bands between 1704–1612 cm^−1^ were assigned to C–O/C=O stretching vibrations of aromatic rings of lignin. A conjugated (Hibbert’s) ketone C=O band at 1699 cm^−1^ might be derived from lignin demethylation of the aromatic methoxy group. Furthermore, 2925–1375 cm^−1^ are the bending vibrations of CH_3_ and CH_2_. So, the presence of abundant surface functional groups such as carboxyl and hydroxyl was confirmed which greatly enhanced the adsorption performance [28,29].

After combining biochar with TiO_2_ NPs, an increase in the relative peak intensity at about 512.32 cm^−1^ is observed for TiO_2_ NC, which is often used for confirmation of vibration of Ti–O. Additionally, 2925–1375 cm^−1^ are the bending vibrations of CH_3_ and CH_2_. The FT-IR spectrum of TiO_2_ NC is shown in Figure 4c.

#### 3.1.4. XRD Diffraction Analysis of TiO_2_, MBM, BC and TiO_2_ NC

**Figure 5:**
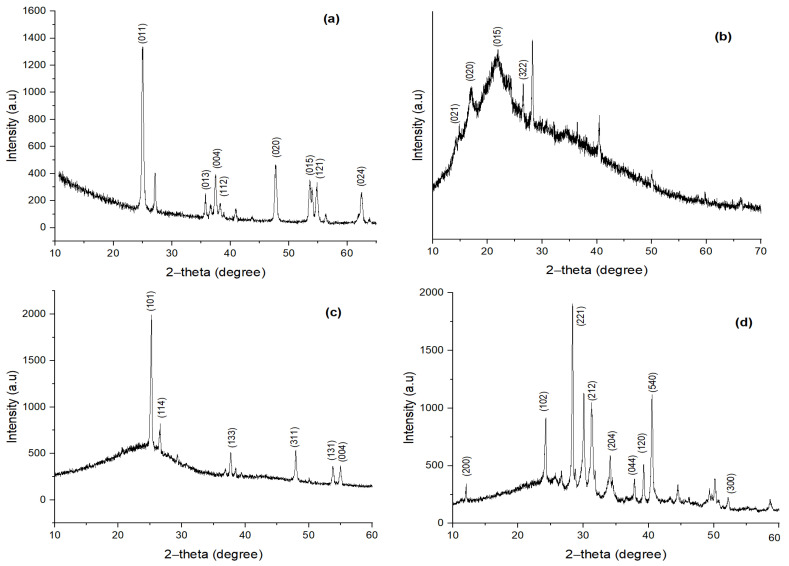
XRD pattern of (**a**) TiO_2_ NP; (**b**) biomass; (**c**) biochar; and (**d**) TiO_2_ NC.

In Figure 5a, strong diffraction peaks are present at 2θ values at 25.19°, 36.82°, 37.65°, 38.44°, 47.92°, 53.73°, 54.93° and 61.96°are associated with planes (011), (013), (004), (112), (020), (015), (121) and (024). These peaks are characteristic of TiO_2_. The X-ray diffraction pattern at 25.19° and 36.82° indicates TiO_2_, all peaks are under the standard spectrum (JCPDS No.: 88–1175 and 84–1286). This diffraction pattern shows that diffraction peak intensity increases with increasing particle size [31], where the peaks of TiO_2_ NC showed the anatase crystalline phase [32]. The average particle size of TiO_2_ NP is found to be 11.73 nm. Lattice parameters are a = 3.78 Å and c = 9.53 Å and the system is tetragonal.

Minor diffraction peaks showing a broad band represent the amorphous structure of MBM. These can be seen in Figure 5b. However, a couple of peaks can be seen at 2θ values. These are 16.5°, 23.6°, 27.4° and 29.5°, associated with planes (021), (020), (015) and (322). These are characteristic of a B-type crystalline structure [33,34]. The calculated particle size through Scherrer’s formula was 0.013.

The diffraction pattern of BC is given in Figure 5c. Strong diffraction peaks are present at 2*θ* values at 16°, 19°, 20.4°, 24.6°, 25.1°, 27°, 33.3°, 38.2° and 42.2°are associated with planes (021), (020), (051), (322), (202), (023), (230), (032) and (112). These signatures relate to crystalline carbon with expanded lattice parameters (carbon with impurities) [35]. The calculated particle size through Scherrer’s formula was 0.932.

The diffraction pattern of TiO_2_ NC is given in Figure 5d. Strong diffraction peaks are present at 2θ values at 25.21°, 26.63°, 36.8°, 47.74°, 54.73° and 55.8°are associated with planes (101), (114), (113), (311), (131) and (044). These strongs peaks confirm the crystalline structure of novel TiO_2_ NC. The calculated particle size through Scherrer’s formula was 0.932.

### 3.2. Optimization of Process Parameters

#### 3.2.1. Effect of pH

**Figure 6:**
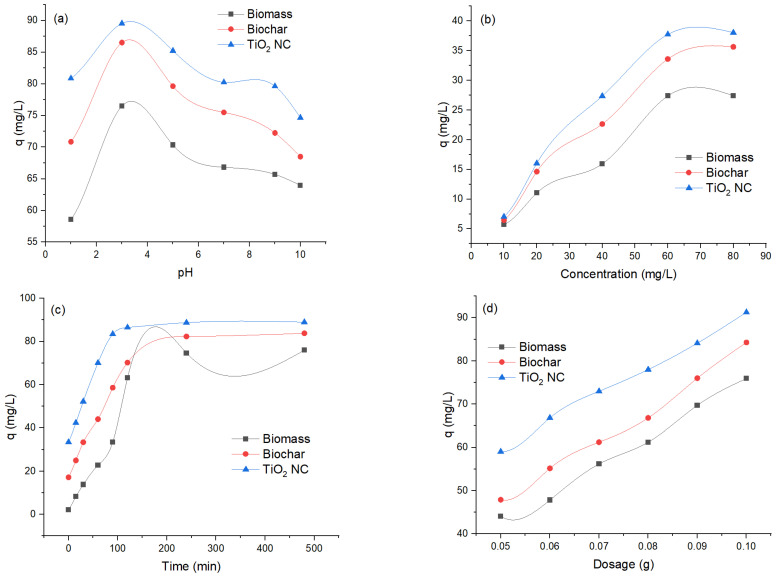
The effect of (**a**) pH; (**b**) concentration; (**c**) contact time; and (**d**) adsorbent dosage for removal of Cr(VI).

The effect of pH on adsorption of Cr(VI) by biosorbents at different pHs ranging from 1 to 10 was observed to evaluate the optimum pH while other variables were kept constant, amount of adsorbent = 0.1 g, temp = 35 °C, shaking speed was 120 rpm and concentration was 100 ppm. In the solution, a decrease in removal efficiency occurs by increasing pH, and maximum removal was observed at 3. Removal efficiency decreases by increasing pH. It was investigated that the adsorbent revealed a comparable binding pattern for Cr(VI). The adsorbent surface becomes negatively charged. It is responsible for electrostatic interaction between adsorbent surface and metals that result in efficient removal of heavy metals. It was demonstrated that different adsorbents showed adsorption of Cr(VI) in comparison with each other. The maximum removal was observed at 92.89 mg/L for TiO_2_ NC and maximum uptake in mg/L of Cr(VI) was decreased to 92.89 (TiO_2_ NC) > 86.51 (BC) > 76.49 (MBM). The effect of pH on adsorption of Cr(VI) by using MBM, B and TiO_2_ NC is graphically represented in Figure 6a.

#### 3.2.2. Effect of Initial Concentration

Initial concentration is responsible for overcoming all barriers to the mass transfer of particles between the solid and liquid phases. The concentration of Cr(VI) solutions varied from 10 to 100 ppm. While other variables were kept constant, amount of adsorbent = 0.1 g, temp = 35 °C, shaking speed was 120 rpm and pH = 3. Results illustrated that percentage removal decreases with an increase in initial concentration. As the amount of adsorbent is the same so competition was negligible at low concentrations but becomes prominent with the increase of concentration of adsorbate molecules. In simple words, the number of adsorption sites was limited at high solution concentrations which hinders the further uptake and hence the percentage removal decreases diffusion. The maximum removal was observed at 93.81 at 10 ppm for TiO_2_ NC maximum uptake capacity in mg/L of Cr(VI) was decreased at 10 ppm as 93.81 (TiO_2_ NC) > 86.34 (BC) > 76.64 (MBM). The effect of initial concentration on adsorption of Cr (VI) by using MBM, BC and TiO_2_ NC is graphically represented in Figure 6b.

#### 3.2.3. Effect of Contact Time

Contact time is a vital factor as it affects the process of adsorption. With the passage of time, more interaction occurs between adsorbent and adsorbate molecules due to which the adsorption rate is increased. In the present work, the effect of contact time on adsorption of Cr(VI) with different adsorbents were studied with a distinct time interval over a range from 15 to 120 min. While other variables were kept constant amount of adsorbent = 0.1 g, temp = 35 °C, shaking speed was 120 rpm, concentration = 100 ppm and pH = 3. The percentage removal efficiency increases as the time increases until equilibrium is achieved.

At the start of the process, there was a higher concentration gradient between adsorbent and heavy metals ions that sped up this process and percentage removal increased by increasing contact time from 0 to 120. At the initial stage of adsorption, a large number of vacant sites were available. As time passes, functional groups present on the surface of the adsorbent take part in the process of adsorption of heavy metals until equilibrium is achieved. The percentage removal increases with an increase in contact time and becomes constant after attaining equilibrium. After equilibrium, the adsorption process may get slow down due to the contribution of other processes, i.e., complexation, saturation of binding sites and micro-precipitation. As the equilibrium was achieved, no more Cr(VI) ions were further adsorbed by increasing contact time because there was no vacant site available resulting in optimization of contact time for the adsorption process. The maximum removal was observed at 88.97 for TiO_2_ NC and maximum uptake in mg/L of chromium was decreased to 88.97 (TiO_2_ NC) > 83.82 (BC) > 75.99 (MBM). The effect of contact time on adsorption of Cr(VI) by using MBM, BC and TiO_2_ NC is graphically represented in Figure 6c.

#### 3.2.4. Effect of Adsorbent Dosage

Cr(VI) removal efficiency as a function of adsorbents dosage was studied. The dose of the adsorbents varied between 0.05 g and 0.1 g, other studied parameters (pH, initial ion concentration and contact time) were kept optimum while temperature and agitation speed were kept at 25 °C and 200 rpm, respectively. Experiments have shown that increasing the dose of the adsorbents increased chromium removal efficiency. This is expected because more binding sites for ions are available at higher doses of adsorbents. These observations described that Cr(VI) adsorption in the wastewater is related to carbon surface area. The adsorbents with the highest surface area are more efficient for removing Cr(VI). The maximum uptake in mg/L of Cr(VI) was decreased to 91.27 (TiO_2_ NC) > 84.27 (BC) > 75.93 (MBM). The effect of adsorbent dosage on adsorption of Cr (VI) by using MBM, BC and TiO_2_ NC is graphically represented in Figure 6d.

#### 3.2.5. Comparison among Different Adsorbents

**Figure 7:**
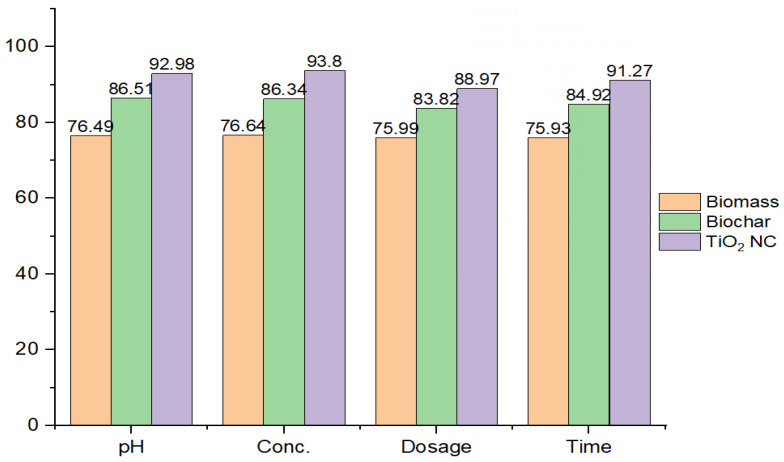
Comparison of adsorption efficiency by prepared adsorbents.

The efficiency of all three adsorbents to remove Cr(VI) from liquid solution was shown in order TiO_2_ NC > BC > MBM. The removal efficiency of TiO_2_NC was found to be highest and respectively followed by BC and MBM. The comparison of uptake capacity of three adsorbents is shown in Figure 7.

Q max results obtained in this work and compared with others reported in the literature, for the same adsorbate, are represented in Table 2.

### 3.3. Adsorption Kinetics

To evaluate the potential rate-controlling step and kinetic mechanism of Cr(VI) biosorption on MBM, BC and TiO_2_ NC pseudo first-order equation of Laguerre, pseudo second-order equation of Ho et al. [41]. Pseudo-1 nature because it only depends on several adsorbate molecules present in an aqueous solution at a definite time. However, the pseudo 2nd-order model describes that lead ions are present in an aqueous solution as well as many free active sites present on the surface of the adsorbent.

#### 3.3.1. Pseudo 1st-Order Model

The pseudo 1st-order regression model presumes that the rate of adsorption and unoccupied sites of Cr(VI) are proportional to each other. The intercept and slopes of the plot of the graph between the values of log(qe-q) time (t) were used for the calculation of pseudo 1st rate constant k1 and the capacity for the equilibrium adsorption. On comparison between pseudo 1st-order and pseudo 2nd-order reactions, it was found that the value of R^2^ in the case of 1st-order is less than that of 2nd-order, so the pseudo 1st-order model was not well fitted. Relatively lower R^2^ values were obtained for the pseudo 1st-order model, R^2^ values for Cr(VI) were MBM (0.70) > BC (0.78) and for TiO_2_ NC (0.68). The theoretically calculated qe(mg/g) did not agree well with experimentally calculated q_exp_. The value of correlation coefficients is lower than 1, most often equal to 0.8. This proves that it is not precise to use this model to conclude the adsorption kinetics by adsorbents MBM, BC and TiO_2_ nanocomposite.

#### 3.3.2. Pseudo 2nd-Order Model

**Figure 8:**
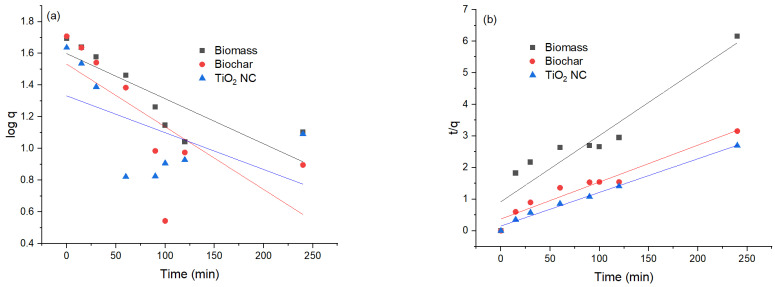
(**a**) Comparison of pseudo 1st-order graph; (**b**) pseudo 2nd-order graph.

Comparison between pseudo 1st-order, pseudo 2nd-order models, and kinetic parameters reveals that adsorption on the MBM, BC and TiO_2_ NC are better fitted in pseudo 2nd-order kinetics than pseudo 1st-order, considering the qe value obtained from pseudo 2nd-order model. It was close to conformation with a value of experimental data value. While the value of qe evaluated from contrasting kinetic models did not comply with the experimental value. This shows that pseudo 1st-order is not satisfactory to explain the mechanism of Cr(VI) biosorption. Evaluation of kinetic data shows that R^2^ values for the pseudo 1st-order and pseudo 2nd-order models are high >0.96 but q^cal^ does not match with q^exp^ to much extent [42]. A comparison of the values obtained from pseudo first-order and pseudo second-order models is given in Table 3 and their graphical representation is in Figure 8a,b.

### 3.4. Equilibrium Modeling

Adsorption isotherms of Cr(VI) from concentrations 10 to 100 ppm were studied with fixed pH 3, and an adsorbent dose of 0.1 g. Freundlich and Langmuir adsorption isotherms were used to define the relationship between adsorbent and adsorbate molecules in an aqueous solution. When there was a changing balance in adsorbate concentration in a large volume of solution with that of boundary, sorption equilibrium was established. To develop the equilibrium data, the primary concentration of Cr(VI) changed. The quantity of adsorbent was kept constant. To get the equilibrium data, the initial concentration of Cr(VI) was changed whereas the number of biosorbents was kept constant. Contact time of 24 h was assured for equilibrium condition of sorption. The Freundlich and Langmuir adsorptions were on constant grade from the isotherm with a correlation coefficient for Cr(VI).

#### 3.4.1. Langmuir Isotherm

Generally, the Langmuir isotherm reveals that the surface of biosorbent and Cr(VI) has equal sites which have the same energy. According to this model, adsorbate molecules do not interact with each other but only interact with the site. Adsorption is restricted to monolayers. It is concluded that once a Cr(VI) ion adsorbs with a site, no more adsorption occurs. When equilibrium was achieved a monolayer appeared [43], which represents the plot of Ce/qe (g/L) versus Ce (mg/L) under various concentrations of Cr(VI) sorption by MBM, BC and TiO_2_ NC. Most of the points in this graph are out of linearity. The values obtained in the Freundlich model are greater than the R^2^ value of the Langmuir model, which suggests the non-applicability of this model in interpreting sorption equilibrium data [44].

#### 3.4.2. Freundlich Isotherm

**Figure 9:**
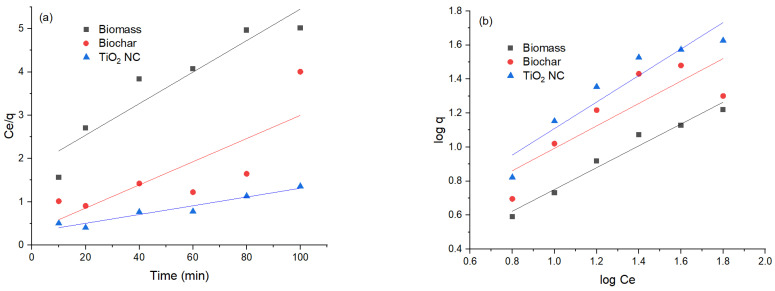
(**a**) Comparison of Langmuir isotherm; (**b**) Freundlich isotherm of lead adsorbed.

It presumes that monolayer adsorption distribution of active sites which are energetically heterogeneous, followed by interaction between biosorbents and adsorbed Cr(VI). The Freundlich isotherm equation is a mathematical expression that describes the adsorption of solute from an adsorbate solution to an adsorbent [44]. A graph is plotted of log qe versus log, this is either an indication that the intercept and graph slopes were used to calculate the value of 1/n or that the adsorption stays continuous or is reduced by raising the concentration of adsorbents. Compared to the Langmuir isotherm plot, this showed more linearity. The value of R^2^ is a result of the correlation coefficient near unity in the Freundlich model. The Freundlich and Langmuir adsorption constants were calculated from the isotherm with a correlation coefficient of Cr(VI). Cr(VI) was introduced to the Langmuir and Freundlich isotherm models. The Langmuir and Freundlich isotherms, however, were proven to be the finest suited and the equilibrium sorption information was well interpreted. A comparison of the values obtained from pseudo first-order and pseudo second-order is given in Table 4, and their graphical representation is in Figure 9a,b.

### 3.5. Response Surface Methodology (RSM)

**Figure 10:**
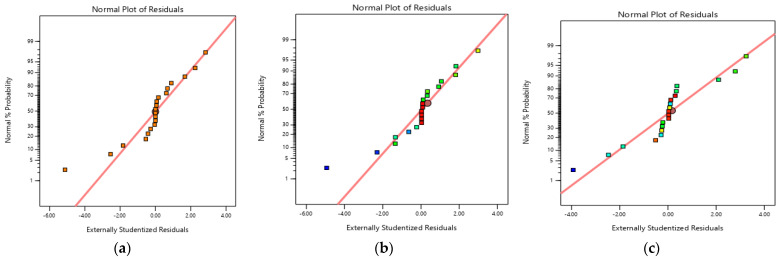
Studentized residuals and normal percentage probability plot for adsorbtion of Cr(VI) by (**a**) biomass; (**b**) biochar; and (**c**) TiO_2_ NC.

The main purpose of any designed experiment is to attain optimum response. RSM is one of the best techniques among all the available techniques. This process mainly helps to understand how test variables affect the selected process response, determine the possible interaction among the test variables, and characterize the combined effect of all test variables on the process response [45]. RSM is used to evaluate the effect of different parameters and for the prediction of targeted response to optimize the reaction. In the present research, interaction and optimization of three important independent variables, i.e., pH (A), initial concentration of Cr(VI) (B) and adsorbent dosage (C) were investigated by using Box–Behnken experimental design for the adsorption of Cr(VI) [46]. The main purpose of the developed model was to analyze the data of the experiment. The studentized residual and normal probability plot is depicted in Figure 10. It can be seen that there was neither any apparent problem with normality nor response transformation in Figure 10 [47]. All the dots lie near the regression line which showed a good correlation between the normal percentage probability and internally studentized residuals.

#### 3.5.1. Fitness of Quadratic Model

The values of coefficient of determination (R^2^) were checked to evaluate the fitness of the quadratic model for Cr(VI) adsorption. R^2^ measures the difference between model and predicted values from the mean value. The value of R^2^ should be close to 1.0 for a model with good prediction efficiency, However, the R^2^ value cannot be so reliable to assess the prediction efficiency of the model because the value of R^2^ is proportional to the number of terms in the model regardless of its statistical significance. So, a comparison of R^2^ with the value of adjusted R^2^ (R^2^adj) values is a better way to assess prediction efficiency, as it reflects the number of factors in the experiment [48]. The value of R^2^adj often decreases if statistically insignificant variables are added. The large difference between the values of R^2^ and R^2^adj shows that some non-significant terms have been added to the model. A close correlation between predicted and experimental values can be indicated by the higher of the R^2^ and R^2^adj values. The obtained values of R^2^ and R^2^adj for MBM, BC and TiO_2_ NC are given in Table 5.

#### 3.5.2. Effect of Independent Variables

**Figure 11:**
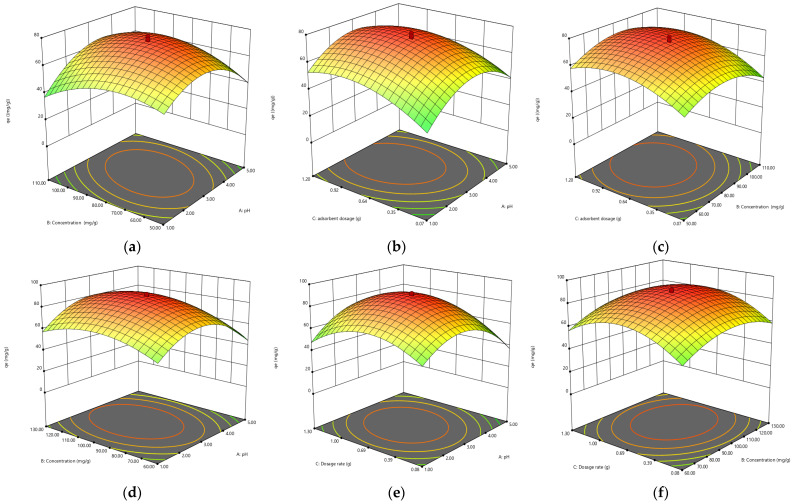

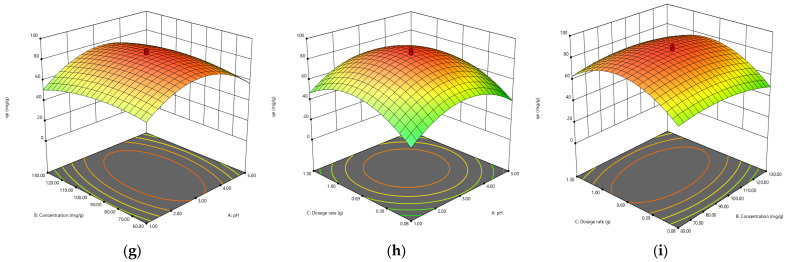
The 3D plots showing the interaction of (**a**) pH × concentration, (**b**) pH × adsorbent dosage, (**c**) concentration × adsorbent dosage by biomass; (**d**) pH × concentration, (**e**) pH × adsorbent dosage, (**f**) concentration × adsorbent dosage by biochar; (**g**) pH × concentration, (**h**) pH × adsorbent dosage, (**i**) concentration × adsorbent dosage by TiO_2_ NC for adsorbtion of Cr(VI).

The combined effect of pH and initial concentration on the adsorption of Cr(VI) was studied by varying the initial concentration from 10–100 ppm and pH range from 1–10, keeping the biosorbent dose constant at 0.1 g/100 mL Cr(VI) solution. The results are presented in a 3D plot in Figure 11. Solution pH greatly affects the Cr(VI) adsorption, as the solution chemistry of adsorbent and adsorbate is greatly affected by pH. Maximum removal was observed at a lower pH range [48].

The combined effect of adsorbent dosage and pH Cr(VI) adsorption was studied by varying the adsorbent dosage from 0.05–0.10 ppm and pH from 1–10, keeping the initial concentration constant. The process of biosorption is significantly affected by the dosage of adsorption. The adsorption capacity (mg/g) of biosorbent decreases with the increase in biosorbent dosage from 0.05 to 0.1 g/100 ppm Cr(VI) solution. It might be due to aggregation or overlapping of active sites, which results in an increase in diffusion path length and a decrease in total surface area available for biosorption of Cr(VI). The highest removal was obtained by using increasing amounts of biosorbents. The results are presented in a 3D plot in Figure 11.

The combined effect of initial concentration solution and biosorbent dose on the Cr(VI) adsorption was studied by varying the biosorbent dosage from 0.05 to 0.1 g and initial adsorbate concentration from 10 to 100 mg/L, keeping the pH adjusted to 3. The results are presented in Figure 11. The biosorption process is strongly controlled by initial concentration. The mass transfer resistance between the aqueous and solid phases is controlled by it. The biosorption of Cr(VI) is affected by both the independent variables. Figure 11 is indicating that the amount of Cr(VI) adsorbed per unit mass of biosorbent is proportional to an increase in the initial concentration of Cr(VI) solution from 10 to 100 mg/L. Maximum removal of Cr(VI) (mg/g) was seen at higher initial concentrations, the reason for this could be the decrease in resistance to uptake the adsorbate from Cr(VI) solution at higher initial concentrations.

## 4. Conclusions

The present research work compared the adsorption efficiency of MBM, BC and biochar-based TiO_2_ NC for Cr (VI). Among these, the synthesized novel nanocomposite has been proven very effective for the removal of heavy metals. In the present research work, potato peel and banana peel were ground to make biomass. Prepared biomass (MBM) was then pyrolyzed to make biochar (BC). Leaves of *Psidium guajava* were utilized to prepare TiO_2_ NP by using titanium precursors. Then prepared biochar was sonicated with TiO_2_ NP to make nanocomposite for better adsorption of heavy metals. Several parameters such as initial concentration of heavy metals, pH, contact time and dosage rate were optimized. The percentage removal of Cr(VI) was investigated, maximum adsorption of Cr(VI) was at pH 3, decrease when the concentration is increased, while percentage removal increases as dosage rate are increased and contact time. The uptake capacity of heavy metals from aqueous solution was in the order biomass (78 mg/L) ˂ biochar (89 mg/L) ˂ TiO_2_ NC (90 mg/L), among them, NC was confirmed with maximum uptake capacity. The Langmuir model better explained the adsorption process of heavy metals as compared to the Freundlich model. Response surface methodology (RSM) was utilized for the optimization of each operational variable.

## Figures and Tables

**Table 1:** Element composition of biomass, TiO_2_ NP & TiO_2_ NC.

Adsorbent	Elements Found	Weight %	Atomic %
Biomass	C	58.6	71.65
	Ca	24.68	10.71
	O	15.74	9.67
	Zr	0.08	3.51
	Ag	0.03	4.22
TiO_2_ NP	Ti	70.5	68.43
	O	29.5	31.85
TiO_2_ NC	Ti	41.69	74.84
	O	39.95	10.84
	Ca	9.65	7.54
	N	4.63	3.65
	C	3.1	2.91
	Zr	0.04	0.05
	Ag	0.61	0.09

**Table 2:** Comparison of adsorption efficiency of different adsorbents.

Adsorbate	Adsorbent	Q Max (mg/L)	References
Cr(VI)	Fe_3_O_4_/pinecones gel beads nanocomposite	212.22	[36]
	MoS2@LDC	198.70	[37]
	OB/ZnO	460.31	[38]
	Polymer-magnetic-algae nanocomposite	144.93	[39]
	Chitosan grafted graphene oxide (CS-GO) nanocomposite	104.16	[40]
Cr(VI)	Mixed biomass (banana and potato peels)	76.49	Present study
	Biochar	86.51	Present study
	TiO_2_/biochar nanocomposite	92.89	Present study

**Table 3:** Comparison of pseudo 1st-order and 2nd-order.

Adsorbate	Adsorbent	Pseudo First-Order			Pseudo Second-Order			
		Qe	k1ad	R^2^	Qexp	qe	k2ad	R^2^
		(mg/g)	(min^−1^)		(mg/g)	(mg/g)	(min^−1^)	
Chromium	Biomass	39.64	−0.0012	0.707	51.67	47.61	0.00048	0.891
	Biochar	45.04	−0.0036	0.789	68.23	85.47	0.00037	0.946
	TiO_2_ NC	23.80	−0.0034	0.684	76.75	94.33	0.00074	0.991

**Table 4:** Comparison of Langmiur and Freundlich models.

Adsorbate	Materials	Langmuir Model			Experimental Value	Freundlich Model			
		X_m_	KL	R^2^	Q	qe	1/*n*	KF	R^2^
		(mg/g)	(L/mg)		q(mg/g)	(mg/g)		(mg/g)	
Chromium	Biomass	23.64	0.02	0.76	16.62	19.48	0.55	−2.14	0.98
	Biochar	26.45	0.08	0.72	19.97	31.94	0.57	−0.9	0.83
	TiO_2_ Nc	59.17	0.04	0.85	42.36	47.22	0.61	−0.52	0.94

**Table 5:** R^2^ and R^2^_adj_ values of biomass, biochar and TiO_2_ NC.

Adsorbent	R^2^	R^2^_adj_
Biomass	0.85	0.74
Biochar	0.91	0.82
TiO_2_ NC	0.98	0.92

## Data Availability

Not applicable.
